# Ophthalmology

**Published:** 1911-03

**Authors:** Cyril H. Walker


### OPHTHALMOLOGY.

**Cataract. Extraction of the lens in its capsule.**—This operation has come still more into prominence in the last year or two. The idea, of course, is not new, and it is now some ten years or more since Major (now Lieut.-Colonel) Smith, with whose name the operation will generally be associated, began to work out the technique necessary for the successful performance of the operation. In his hospital at Jalandhar he has extracted more than 22,000 cataractous lenses in this manner. It is probable that any of the recognised operations, in the hands of a skilful operator with such vast experience, would have given excellent results. The advantages claimed for Colonel Smith's method are the almost complete elimination of iritis, the avoidance of a troublesome and sometimes dangerous operation for the division of the secondary membrane, and above all the possibility of operation in any stage of an immature cataract. The operation is applicable only to adults, and should not be attempted before the age of thirty, as until that age the adhesion of the posterior capsule of the lens to the hyaloid is so intimate that vitreous is certain to be lost. It is this dread of losing vitreous that has hitherto deterred most European and American operators from adopting the method. The fear was that although the eye might survive the operation, even though a considerable quantity of vitreous might be lost, yet in the long run the eye would not "wear well." To test this objection Lister[1] has undertaken a research in the hospital records at Jalandhar. Many thousands of cases were investigated, and wherever the escape of vitreous was noted the patient was written to. Ninety-five presented themselves for examination. The different periods since the date of operation ranged from six months to nine years. The average 3.7 years. Lister concludes that the amount of vitreous lost does not materially affect the ultimate result. It is specially important to note that detachment of the retina, so generally dreaded after escape of vitreous, did not occur in a single instance. It therefore appears probable that it is a serious complication only when the eye is hampered with the debris and capsule of the lens.

Very briefly, the chief features of Colonel Smith's technique are as follows. After the section of the cornea and the iridectomy the speculum is removed, and the eye entrusted to a carefully trained assistant, who holds the lids apart with his fingers, and at the same time hooks up the upper lid with a strabismus hook. This removes all possibility of the patient exerting pressure on the globe by means of his lids or orbicularis. The operator then tries to rupture the suspensory ligament by pressing backwards with the point of a strabismus hook near the lower corneal margin. As soon as the edge of the lens presents in the wound, it is followed upwards by the hook. If the capsule ruptures an attempt must be made to extract the remains of it with forceps. If vitreous presents in the wound the delivery of the lens must be assisted with the spatula or vectis.

It appears that a better trained assistant is required for this than for the ordinary operation, and that the operator himself requires rather more practice in order to do justice to the operation; but in any case it seems likely to mark a distinct advance in ophthalmic surgery, and to render a decided service to humanity.

An important criticism of Smith's operation is furnished by Major Kilkelly in the *Indian Medical Gazette* of May, 1910. At his request Colonel Smith operated on twenty-three cases in Bombay. The patients were kept in the hospital for periods varying from fifteen to twenty-seven days. The vision on discharge was only in one case better than $\frac{6}{18}$. In five cases

[1] *Arch. Ophthal.*, 1910, xxxix. 1.

the capsule ruptured, vitreous escaped in two cases. In five cases there was incarceration of the iris in the angles of the wound, and seven suffered from iritis. Though he has done the operation in more than 600 cases, Major Kilkelly has abandoned it, and the results of these twenty-three cases confirm him in his decision. Time only will show in what instances the operation is applicable.

**Arsenic compounds and optic atrophy.**—Now that organic compounds of arsenic are being used in the treatment of many protozoal diseases, such as syphilis, malaria, recurrent fever and the like, the possible risk attending their use must not be forgotten. Koch reported twenty-three cases of optic atrophy following the use of atoxyl in patients suffering from sleeping sickness. Schirmer [1] records a case in a man of 58 after two injections of atoxyl a week for six months, the total amount given being 9.9 grammes. After the treatment had been persisted in for about two months the vision began to fail. Twelve months later he was totally blind. Similarly a man aged 73 suffering from psoriasis became totally blind about four months after six injections of arsacetin given in a period of four weeks. Both these cases occurred in diseases never known to produce optic atrophy. Another typical example is quoted by Coppez. [2] A man of 54 with inherited syphilis, interstitial nephritis and cardiac lesions, received a series of ten injections of 5 centigrammes each of atoxyl. The sight began to fail after the first injection, and blindness was complete after the fifth; but this was looked upon as an indication for pushing the remedy. It was not until five weeks after the onset of the blindness that pallor of the discs became apparent. No recovery of vision occurred. The total number of such cases recorded approaches forty, or is even more. The clinical phenomena resemble quinine blindness, but in this latter partial recovery is the rule. Dimness of sight and flashes of light appear usually to be the earliest symptoms. At first there are no ophthalmoscopic changes, but almost invariably the visual fields are contracted.

Ehrlich's "606" appears to be free from the dangers attending the use of atoxyl. An epitome of the experience of Schanz, of Munich, [3] suggests that it was of some use in five cases of interstitial keratitis. In one case of early syphilitic iritis one injection was followed by the disappearance of the inflammatory signs and giving way of the synechiæ. In one week after a single dose, and with the use of atropin, the eye had recovered. It has also proved of marked use in syphilitic papillitis. It is early to predict, but hitherto no case

[1] *Arch. Ophthal.*, 1910, xxxix. 456.
[2] *La Clinique Ophthal.*, June, 1909.
[3] *Ophthal. Rev.*, xxx. 25.

has been recorded in which "606" has been followed by serious damage to sight.

**Trachoma.**—Since Greef and others demonstrated the minute "trachoma bodies" in pairs or dumb-bell forms some two years ago, similar if not identical bodies have been found by several observers in blenorrhœa, both conjunctival and genital. Herzog traces a close connection between the gonococcus and trachoma bodies. He reports an early case of trachoma, with an unusual number of cell-inclusions, many cells containing paired organisms, like gonococci, but smaller. He regards them as possibly being transition forms. The examination of another case of acute conjunctival blenorrhœa showed typical gonococci in profusion. After six days' energetic treatment there were no gonococci, but numerous cell-inclusions were found for the first time, exactly like trachoma bodies. Four weeks later the microscopical findings were negative. Further proof of this association by cultures is lacking, but it is significant that apart from trachoma these characteristic formations have only been found in cases of gonorrhœal origin.

**The action of astringents in conjunctivitis.**—Formerly the accepted theory was that astringents caused a superficial slough, and that this carried away the majority of the diseased cells. Later the "antiseptic" theory led to douching of the conjunctiva on the supposition that the drug acted directly on the microbes. Schneider concludes that there is no bactericidal substance in normal lachrymal or conjunctival secretion, but that the application of even 1 in 1,000 solution of nitrate of silver causes the formation of "leukines" in the conjunctival secretions. The same result is produced by protargol and sulphate of zinc. After the instillation leucocytes pass into the conjunctival sac and give off leukines, and it is upon the production of leukines that the curative power of astringents depends.

CYRIL H. WALKER.

